# Discovery of potent, novel, non-toxic anti-malarial compounds via quantum modelling, virtual screening and *in vitro *experimental validation

**DOI:** 10.1186/1475-2875-10-274

**Published:** 2011-09-20

**Authors:** David J Sullivan, Nikola Kaludov, Martin N Martinov

**Affiliations:** 1 Department of Molecular Microbiology and Immunology, Johns Hopkins Bloomberg School of Public Health, Baltimore, Maryland, USA; 2 Gradient Biomodeling, LLC, Park City, Utah USA

## Abstract

**Background:**

Developing resistance towards existing anti-malarial therapies emphasize the urgent need for new therapeutic options. Additionally, many malaria drugs in use today have high toxicity and low therapeutic indices. Gradient Biomodeling, LLC has developed a quantum-model search technology that uses quantum similarity and does not depend explicitly on chemical structure, as molecules are rigorously described in fundamental quantum attributes related to individual pharmacological properties. Therapeutic activity, as well as toxicity and other essential properties can be analysed and optimized simultaneously, independently of one another. Such methodology is suitable for a search of novel, non-toxic, active anti-malarial compounds.

**Methods:**

A set of innovative algorithms is used for the fast calculation and interpretation of electron-density attributes of molecular structures at the quantum level for rapid discovery of prospective pharmaceuticals. Potency and efficacy, as well as additional physicochemical, metabolic, pharmacokinetic, safety, permeability and other properties were characterized by the procedure. Once quantum models are developed and experimentally validated, the methodology provides a straightforward implementation for lead discovery, compound optimizzation and *de novo *molecular design.

**Results:**

Starting with a diverse training set of 26 well-known anti-malarial agents combined with 1730 moderately active and inactive molecules, novel compounds that have strong anti-malarial activity, low cytotoxicity and structural dissimilarity from the training set were discovered and experimentally validated. Twelve compounds were identified *in silico *and tested *in vitro*; eight of them showed anti-malarial activity (IC50 ≤ 10 μM), with six being very effective (IC50 ≤ 1 μM), and four exhibiting low nanomolar potency. The most active compounds were also tested for mammalian cytotoxicity and found to be non-toxic, with a therapeutic index of more than 6,900 for the most active compound.

**Conclusions:**

Gradient's metric modelling approach and electron-density molecular representations can be powerful tools in the discovery and design of novel anti-malarial compounds. Since the quantum models are agnostic of the particular biological target, the technology can account for different mechanisms of action and be used for *de novo *design of small molecules with activity against not only the asexual phase of the malaria parasite, but also against the liver stage of the parasite development, which may lead to true causal prophylaxis.

## Background

Malaria is one of the most widespread infectious diseases of our time. Even though the global malaria map has been shrinking over the past 50 years, more people are at risk of suffering from malaria today than at any other time in history-close to 40% of the world's population live in countries where the disease is endemic and nearly 247 million people suffer from the disease every year [1]. Malaria is caused by protozoan parasites of the genus *Plasmodium *that infect and destroy red blood cells, leading to fever, severe anaemia, cerebral malaria and, if untreated, death. *P. falciparum *is the dominant species in sub-Saharan Africa, and is responsible for almost one million deaths each year. The disease burden is heaviest in African pregnant women and children under five years of age, who have frequent attacks and weak immunological protection. The global fight to control malaria requires a multi-faceted approach [2]. At present, a wide range of effective tools exists, including insecticide and larvicide spraying, the use of insecticide-impregnated bed nets to protect against infection by mosquitoes, and medicines to both treat the infection and prevent it in pregnant women and in young children [3-5]. However, long-term prophylaxis by vaccination has been especially challenging as the parasite has various sophisticated mechanisms to avoid the host immune system and no approved vaccine is currently available [6]. Even with all available strategies combined, a substantial number of patients will suffer from this disease over the coming decades. Due to emerging drug resistance, new medicines are needed to treat malaria episodes, mainly targeting the asexual blood stages of *P. falciparum *[7]. Blocking the transmission of the parasite by the mosquito vector and, in the case of *P. vivax *infections, targeting the dormant liver stage of the parasite, are other important steps towards eradication of the disease [8,9].

Traditional experimental methods for high-throughput screening and identification of novel compounds with activity against malaria targets are rarely effective for new therapeutics reaching clinical use. Despite considerable investments of resources and continuous improvements of these methods [10], many false positive hits arise and require further effort to triage and validate the results [11]. Increased size and complexity of molecular screening libraries often reduce the chance of finding leads among randomly chosen ligands [12] because of practical limitations associated with synthesis and testing of additional compounds with low probability of matching the requirements of the pharmaceutical target. Therefore, it is not surprising that high-throughput screening efforts frequently fail to identify suitable hit compounds, particularly against targets have not been studied previously. In attempts to improve hit rates, screening efforts are often enhanced by traditional structure-based computational methods [13,14], without much success [15-17].

In contrast, Gradient's methodology did not use any prior, explicit knowledge of the malaria protein targets. Metric modelling considers the compounds of interest as quantum objects, without explicit dependence on their particular chemical structure. On a theoretical level, these quantum properties serve as powerful descriptors for molecular modelling, compound identification, optimization and *de novo *design. The computational platform determines essential, rigorous, easily computable molecular attributes related to chemical activity. These attributes are derived from a special representation of quantum fields. Their well-defined mathematical characteristics afford systematic theoretical treatment and property prediction with methods that would otherwise be computationally impossible. Specialized machine-learning algorithms with fuzzy decision-making protocols are applied to identify both active compounds and the corresponding quantum features of chemical and biological interest. Combined with the underlying modelling architecture, the algorithms also provide mechanistic hypothesis for the modelled interactions. Since structurally dissimilar compounds can be similar on a quantum level, this process is particularly good at identifying chemically novel compounds that have significant potency against the target.

## Methods

### Platform implementation for identification of existing molecules with anti-malarial activity

The first step for the generation of a model is the creation of a set of predictive quantum filters. A quantum filter is a software module that uses quantum attributes of molecules to predict their activity, or any other observable property. These filters are created by the use of a training set of compounds with known activity against the target, a crystal structure of the target, or other data related to the desired property. The structural data is first used to compute the quantum components of the involved molecular systems. Then, fuzzy machine-learning algorithms are used to classify the target property with respect to these pre-computed quantum objects. Quantum components controlling the target properties are then used for property prediction of prospective molecules. The resulting quantum filter is experimentally validated by virtual high-throughput screening of a comprehensive database of pre-computed commercially available compounds, identification of potential active molecules and testing them *in vitro *for activity in the appropriate biological assay.

### Structure representation-localized electron-density descriptors for molecular modeling

Well-defined chemical subsystems, together with their associated local, spatially-resolved properties, are very useful in drug discovery [18]. On a theoretical level, these properties serve as powerful descriptors for molecular modelling and design. Notions from Density Functional Theory and Topological Theory of Atoms in Molecules can be combined to rigorously define and compute a complete set of such localized, electron-density descriptors.

In general, Non-Relativistic Quantum Mechanics (QM) provides the proper level of physical theory for treatment of molecular and bio-molecular systems [19,20]. However, many intuitive chemical concepts are not directly related to the corresponding wave function [21], a state-vector in Hilbert space, which is difficult to partition into chemically meaningful subsystems [22-25].

Density Functional Theory (DFT) [26,27] provides a systematic framework for inferring chemistry-related information from QM calculations. This is achieved through the use of the electron density, ρ(**r**), a real, nonnegative Cartesian function connected to the *N*-electron molecular wave function ψ by

$$\rho \left(r\right)=\int |\psi \left(x,x_{1},\ldots ,x_{N-1}{|}^{2}dsdx_{1}\ldots dx_{N-1}.,\right)$$

where **x **= {*s*, **r**} is the four-dimensional spin-spatial coordinate. As the famous Hohenberg-Kohn theorem [28] shows, ρ(**r**) determines all ground-state properties of the entire system, including its chemical and biochemical features.

Furthermore, the Topological Theory of Atoms in Molecules (AIM) [29,30] uses ρ(**r**) to partition molecules into precise atomic subsystems. These atomic subsystems are bounded by zero-flux surfaces *S*, which obey the equation

$$\forall r\in S\;n\left(r\right)∙\nabla \rho \left(r\right)=0,$$

where **n**(**r**) is the vector normal to *S *at **r **and ρ(**r**) is the corresponding electron density.

It is natural to combine DFT and AIM, together with their respective computational algorithms, in a single formalism for studying local molecular properties from first principles. This formalism has yielded meaningful interpretations of many general chemical concepts, such as energy partitioning [31], atomic softness [32], electronegativity equalization [33], atomic reactivity indices [34], etc. Augmented with the electrostatic potential, this electron density-based methodology has been applied to quantitative structure-activity relationship studies [35,36]. It also produced the molecular descriptors employed in the modelling effort described here. Importantly, when applied as descriptors, these electron-density transforms define a proper metric (molecular similarity measure) in the modelling space and allow the use of rigorous mathematical techniques.

### Modelling architecture-fuzzy decision networks

Molecular modelling is a multi-step process:

$$\left\{Si,Pi\right\}\to \left\{D_{i,j}\left(S\right),P_{i}\right\}\to P\left[D\left(S\right)\right]$$

The starting point, {*S_i_, P_i_*}, called a training set, is a set of molecular structures *S_i_*for which a particular property of interest *P *has been measured. In the first step, descriptor calculation, every structure is reduced to some form, typically a list of real numbers {*D_j_*}, which can be modelled statistically. The second step, actual modelling, attempts to find a model-a general mapping between property *P *and structure *S *through descriptors *D*. If successful, the model would have predictive power that can be applied to structures for which no measurement exists. Naturally, the predictive power of the model depends on the quality (accuracy, diversity, etc.) of the training set as well as descriptor properties and modelling architecture.

Both powerful descriptors and proper modelling architecture are crucial for successful molecular modelling and compound discovery. Ideally, the modelling architecture should be chosen in accordance with the underlying fundamental processes of the system [37], and not with the type of available numerical data. Complex biochemical interactions involve local attributes of distinct and diverse molecular structures, which are best modelled with discrete combinatorial methods rather than continuous multivariate techniques. Still, inherent weaknesses of traditional molecular descriptors require the use of such continuous multivariate techniques [38]. As sophisticated as some of these techniques are, they cannot always compensate for the shortcomings of the underlying molecular-structure representations.

**Figure 1:**
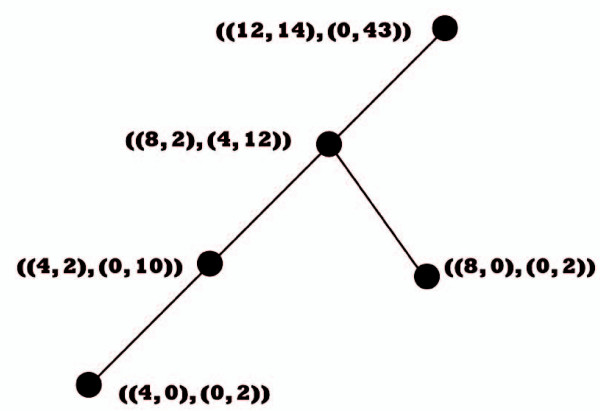
**Fuzzy decisions tree and network**. Quantum attributes are used to represent molecular systems. These attributes define a specific, relevant metric in the modelling structure space and thus allow the proper use of rigorous mathematical techniques. Also, they have strong relationships with chemical features controlling molecular interactions, and well-defined physical properties are often related to a single attribute. In its simplest implementation, the modelling algorithm produces a fuzzy decision tree, which can be generalized to a more powerful fuzzy decision network. Each tree node corresponds to a single attribute (interaction constraint). The attribute explaining most data variance occupies the highest node. In a fully resolved decision tree, terminal nodes contain only either active or inactive molecules. Further, each terminal node is fully characterized statistically by associated confidence intervals and other parameters. A decision network provides a complete characterization of the interaction patterns found within the modelling data.

A straightforward machine-learning algorithm using fuzzy-logic decisions easily discovers the relationship between quantum components and specific interaction patterns. In its simplest implementation, the modelling algorithm produces a model (Figure 1) in the form of a fuzzy decision tree [39,40]. Each tree node corresponds to a single descriptor (interaction constraint). In a fully resolved decision tree, terminal nodes contain only either active or inactive molecules. Furthermore, each terminal node is fully characterized statistically-if a molecule belongs to it, the prediction is qualified by associated confidence intervals and other statistical parameters. A model in the form of a decision tree is easy to interpret. Each tree path that contains an active terminal node also contains a set of nodes (quantum components) that define the interaction pattern common to all training-set molecules belonging to this terminal. The fuzzy decision tree formalism can be generalized [41] to more powerful fuzzy decision algorithms. Given a diverse training set of structures with known inhibition, the modelling effort produces a decision network characterizing all present interaction patterns in terms of activity-controlling descriptors, which can be visualized [36].

### Modelling data and *in silico *filters

**Figure 2:**
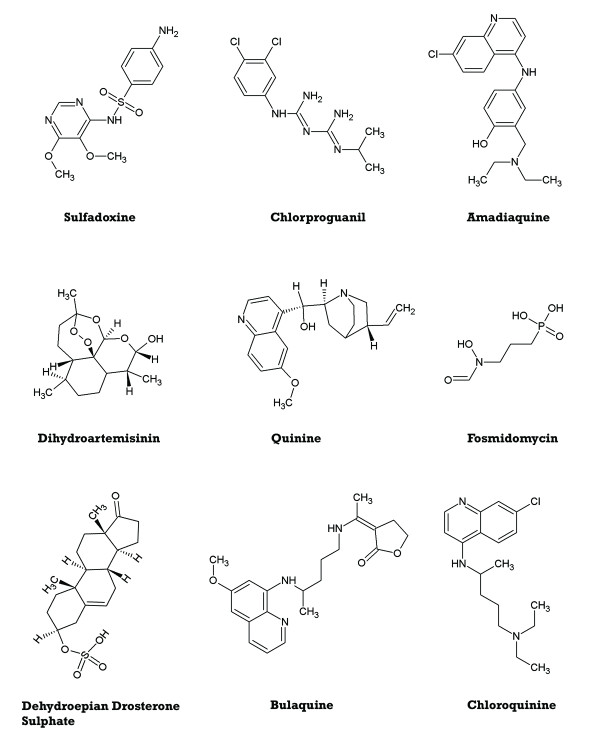
**Anti-malarial training set example structures**. The core of the training set used to create the anti-malarial filter consists of 26 known anti-malarial compounds (full list in Table 1).

Three separate *in silico *models were created and then applied as screens for anti-malarial compound identification. The anti-malarial model was based on a training set consisting of 26 known anti-malarial compounds (Table 1, Figure 2) and *in vitro *data generated at the Johns Hopkins Malaria Research Institute of 1,730 FDA drugs. The *in vitro *data consisted of single-point measurements of anti-malarial percent inhibition at 10 μM concentration. Since the modelling resolution is directly related to the input data accuracy, the predictive threshold of the model was predetermined by the training set at the same 10 μM value. The training set included multiple compounds with no *P. falciparum *inhibition, which is important to define negative interaction constraints in quantum terms. The chemical-diversity screen was developed and applied to assure that the identified compounds are novel and chemically different than the 26 known anti-malarials. A number of commonly accepted theoretical measures of molecular similarity [42] were considered to estimate how novel the proposed compounds are. These include Tanimoto coefficients [43] based on pharmacological functional groups or compound fragments [44,45], as well as chemical diversity measures derived from electron density considerations [39]. Once computed, these indices are used to create point-to-set distance metrics [46], which determine the dissimilarity of the considered structure from the 26 known active molecules. Finally, to create filters accounting for low cytotoxicity, the publicly available data from the National Center for Computational Toxicology [47] and other sources was used [48].

**Table 1:** Anti-malarial training set-full list of molecules

sulphadoxine	amodiaquine	atovaquone	mefloquine
pyrimethamine	artesunate	proguanil	pyronaridine

chlorproguanil	dihydroartemisinine	azithromycin	artemether

Dapsone	piperaquine	quinine	lumefantrine

Dihydroartemisinin	chloroquine	bulaquine	tafenoquine

Trimethoprim	sulphamethoxazole	fosmidomycin	clindamycin

Artemotil	dehydroepian drosterone sulphate	-	-

### The database

A compound database of commercially available molecules was compiled for the virtual screening part of this experiment. A total of about 5.8 million structures were included by incorporating compounds from multiple sources like Enamine, ChemBridge, LifeChemicals, ChemDiv, TimTec, the National Cancer Institute, etc. All molecules were pre-computed and stored in quantum binary form for virtual screening purposes.

### *In vitro *anti-malarial activity assay

The *in vitro *anti-malarial activity was measured using the [3H]-hypoxanthine incorporation assay [49] with various strains of *P. falciparum *(Roche). Results were expressed as the concentration corresponding to 50% inhibition. The anti-malarial assays were performed at the Swiss Tropical Institute, Basel, Switzerland.

### Toxicity assay

Toxicity was determined by using the colorimetric 3-(4,5-dimethylthiazol-2-yl)-2,5-diphenyltetrazoliumbromide (MTT) assay [50]. The toxicity assays were performed at the Swiss Tropical Institute, Basel, Switzerland.

## Results and Discussion

Conventional drug discovery implies a slow, incremental search for chemicals structurally similar to known active compounds. This process is inherently limited and rarely finds molecules having at once all the required properties of a successful drug. Chemical structure alone does not provide adequate description of molecular interactions, which are quantum in nature. Without quantum science any understanding of molecular interactions is incomplete. In theory, chemistry and biology can be fully derived from quantum mechanics [19,20]. It was hypothesized that quantum representation of a small, diverse set of known anti-malarial compounds can be used identify *in silico *novel, non-toxic molecules that inhibit *P. falciparum*. Starting with the training set of the 26 known anti-malarials and the 1730 FDA drugs with malaria activity screened at 10 μM, a quantum anti-malarial model was constructed. The model produced 12 quantum components positively correlated to activity. Each of these quantum components can be expressed by multiple chemical substructures. In addition to the 12 active quantum components, the model produced more than 20 quantum components with negative correlation to malaria action. This translated to detrimental chemical substructures which were also used in the subsequent *in silico *screening process. Additional filters generated from the data from the National Center for Computational Toxicology were used to ensure that the selected compounds were non-toxic.

**Figure 3:**
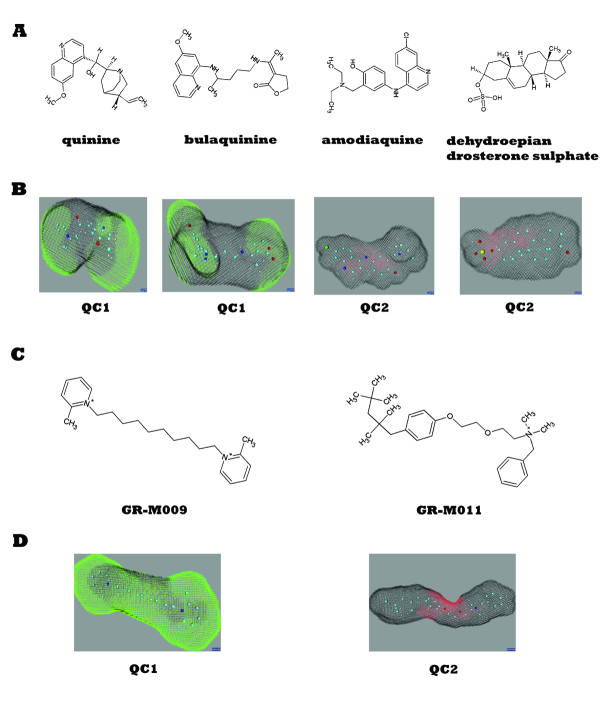
**Quantum anti-malarial model**. Starting with known anti-malarial drugs (**A**), quantum components (QCs) that control anti-malarial activity were identified (red and green shaded areas). Dissimilar nuclear arrangements in active molecules can have similar anti-malarial electron density transforms (EDTs). For example, even though the red-shaded area illustrated as QC2 in panel A is comprised of different atoms in a different chemical substructure, the algorithms calculated their anti-malarial EDTs to be similar to one another. The QCs (**B**) were calculated and visualized as described in Materials and Methods. The subsequent virtual screen identified novel compounds (**C**) predicted to be active against P. falciparum based on these pre-computed anti-malarial QCs, i.e., it discovered molecules with novel nuclear arrangements that carry the same anti-malarial QCs (**D**). Containing two symmetrical quantum components (2 × QC1) which encompass the entire molecule, GR-M009 is the most active novel compound, while the less active GR-M011 contains only one quantum component (QC2). Red dots represent oxygen atoms, dark blue dots represent nitrogen atoms, light blue dots represent carbon atoms, and yellow dots represent sulphur atoms.

**Figure 4:**
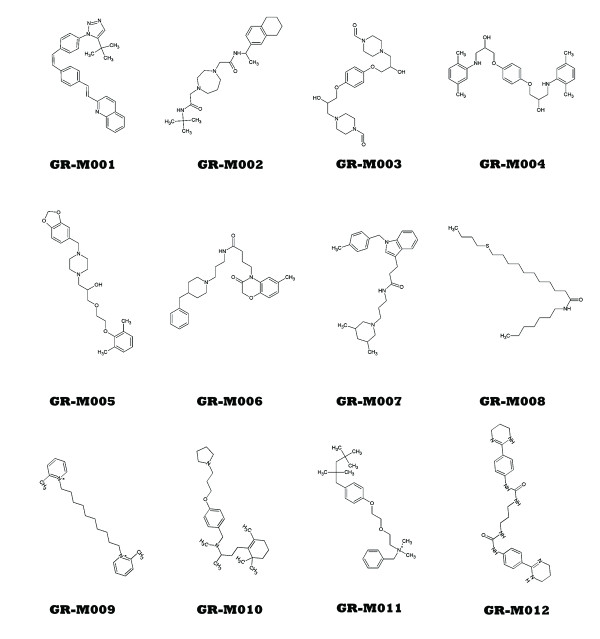
**Novel anti-malarial compounds identified through quantum metric modelling**.

Since the modelling procedure employed this diverse training set of structures with known activity (Table 1, Figure 2), it produced a model in the form of a decision network characterising multiple anti-malaria interaction patterns. These interactions were defined in terms of activity-controlling quantum attributes, which were visualized by projection on corresponding Cartesian molecular surfaces (Figure 3). Importantly, the same quantum attribute can be found on chemically dissimilar molecules, which enables discovery of novel molecules with anti-malarial activity. Quantum attributes as shown in Figure 3 were used to construct the malaria-specific *in silico *filter. Together with the quantum toxicity filter and the chemical diversity filter described in the Materials and Methods, it was used in a virtual search for novel, non-toxic anti-malarial compounds. The virtual screen identified a number of molecules from the quantum database and rank-ordered them according to their quantum anti-malarial attributes. Based on commercial availability, twelve of the top 25 rank-ordered compounds were obtained for *in vitro *validation in both anti- *P*. *falciparum *and mammalian cytotoxicity assays. The *in vitro *results are presented at Table 2. All tested molecules are novel, with low structural similarity (average Tanimoto coefficient < 0.2) to the known drugs used in our training set (Figure 4). Eight of the twelve showed anti-malarial activity at or below the modelling threshold (IC50 = 10 μM), with six being very effective (IC50 ≤ 1 μM). Four compounds exhibited potency in the low nanomolar range (IC50s of 27nM, 185nM, 328nM and 332nM, respectively). The toxicity of the six most active molecules was also measured (Table 2), and their respective therapeutic indices (ratios of anti-malarial activity to mammalian cytotoxicity) were calculated. The most potent of the tested compounds has an index greater than 6900. In contrast, most malaria drugs in use today have much lower therapeutic indices [51]. These results exceed conventional state-of-the-art computational methods and are not subject to the limitations of popular docking programs [52]. For example, these findings substantially outperform a recent malaria study [17,53] not only in success rate of compound discovery (75%), but in speed and need for computational resources as well. The research described here confirms the ability of the quantum-similarity platform to generate anti-malarial compounds that are simultaneously active, novel and non-toxic-the three most important characteristics of an effective therapy for this disease. Once validated, the quantum anti-malarial components discovered by this approach can be employed in straightforward *de novo *design of new chemical entities possessing these three features as well as all other ADME/Tox properties required for successful anti-malarial therapeutics.

**Table 2:** Summary of anti-malarial activity and cytotoxicity

Compound	Average Anti-malarial Activity**(ng/ml)	IC50 (μM)	Average Cytotoxicity** (ng/ml)	Therapeutic Index	Average Tanimoto Coefficient
GR-M001	> 10'000*	> 20.0	ND	NA	0.170

GR-M002	5381	12.54	ND	NA	0.162

GR-M003	> 10'000	> 20.0	ND	NA	0.148

GR-M004	4778	10.28	ND	NA	0.175

GR-M005	9736	18.91	ND	NA	0.147

GR-M006	1634	3.520	ND	NA	0.175

GR-M007	446	0.999	10420	23	0.186

GR-M008	382	1.027	> 90'000	> 236	0.124

GR-M009	13	0.027	> 90'000	> 6923	0.159

GR-M010	172	0.332	4860	28	0.183

GR-M011	86	0.185	3250	38	0.159

GR-M012	168*	0.328	89770	533	0.145

Anti-malarial control (chloroquine)	4.2	0.013	NA	~2	ND

Cytotoxicity control (Podophyllotoxin)	NA	NA	9.5	NA	NA

*Not fully soluble in DMSO

**Data from two independent assays

ND: not determined

NA: not applicable

Given the growing resistance of the malaria parasite, the ability to discover new classes of active, safe molecules will be essential in the search for anti-malarial agents. Furthermore, since quantum features can be defined for compounds known to impact different stages of the parasite life cycle, the methodology could provide, for instance, an opportunity to identify alternative *P. vivax *hypnozoitocidals by the use of primaquine, tafenoquine and pamaquine in the training set [7,9]. This is an extremely difficult and demanding area of biology and there is a great need for alternative radical cures without side effects. Finally, this computational platform opens the possibility of exploring novel chemical spaces and specifically and accurately targeting elusive, hard to modulate protein-protein interactions previously considered unapproachable by current discovery methods.

## Conclusions

To summarize, after starting from a training set of 26 known anti-malarial drugs and a collection of 1730 FDA drugs, several novel, chemically different molecules with high potency and low toxicity were identified and experimentally validated by testing only 12 compounds. The computational work was performed in less than a month on a single computer. Together with the experimental validation, the whole process took less than four months and required significantly smaller resources than similar drug discovery efforts. Gradient's innovative approach significantly reduces the time and cost needed to generate pre-clinical drug candidates and greatly improves the chances to discover and develop a true causal anti-malarial prophylaxis therapy.

## Competing interests

The core quantum technologies being used by Gradient Biomodeling, LLC, were developed and are owned by Martin N. Martinov, PhD, the chief scientist and managing partner of Gradient.

## Authors' contributions

MNM and DS conceived and designed the study. MNM performed the quantum analysis. MM, NK and DS analysed the data. MNM and NK produced figures and tables. MNM, NK and DS drafted the manuscript. All authors read and approved the final manuscript.
